# Anal Cancer Incidence and Survival: Comparing the Greater San-Francisco Bay Area to Other SEER Cancer Registries

**DOI:** 10.1371/journal.pone.0058919

**Published:** 2013-03-06

**Authors:** E. Susan Amirian, Paul A. Fickey, Michael E. Scheurer, Elizabeth Y. Chiao

**Affiliations:** 1 Dan L. Duncan Cancer Center, Baylor College of Medicine, Houston, Texas, United States of America; 2 Department of Pediatrics, Baylor College of Medicine, Houston, Texas, United States of America; 3 Department of Medicine, Baylor College of Medicine, Houston, Texas, United States of America; Memorial University of Newfoundland, Canada

## Abstract

The incidence of squamous cell carcinoma of the anus, anal canal, and anorectum (SCCA) has increased over time. However, there are still no national guidelines on screening for SCCA among high-risk populations. Providers at University of California, San Francisco have been at the forefront of providing anal dysplasia screening. To determine whether such a screening program allows for earlier detection of abnormalities and consequently, improves patient survival, we conducted an ecological study using data from the Surveillance, Epidemiology, and End Results (SEER) program to compare the San Francisco-Oakland catchment area (SF-O) to other SEER sites where routine screening has not been as accessible. Cox regression models were utilized to assess the impact of residing in the SF-O region, versus other SEER sites, on cause-specific mortality hazard. Logistic regression was used to determine if site was associated with the probability of having an *in situ* versus invasive tumor among SCCA cases. All analyses were stratified on calendar time (1985–1995 and 1996–2008) to compare differences pre- and post- highly active anti-retroviral therapy. Among SCCA cases, being reported by the SF-O registry was associated with a four fold higher probability of having an in situ tumor (rather than an invasive tumor) [95% CI: 3.48–4.61], compared to sites outside of California, between 1996 and 2008. Cases reported from the SF-O region between 1996 and 2008 had a 39% lower mortality risk than those reported from registries outside California (95% CI: 0.51–0.72). However, there was no decrease in the rate of invasive SCCA over this period. This is the first ecological study to evaluate whether access to anal cancer screening programs may help improve patient survival by allowing for earlier detection of lesions. Our results imply that routine screening programs may help detect SCCA at an earlier stage and thus, potentially impact patient survival.

## Introduction

Although rare in the U.S., squamous cell carcinoma of the anal canal comprises approximately 2% of all intestinal cancers globally [1], [2]. Risk factors include infection with human papillomavirus (HPV) and immunosuppression [3]. In the U.S. and other developed countries, the incidence of squamous cell carcinoma of the anus, anal canal, and anorectum (SCCA) has continually increased over the last several decades [4], [5], [6], [7]. SCCA incidence is higher among women [8], HIV-positive populations [9], and men who have sex with men (MSM) [7], [9], [10]. The highest risk population is HIV-infected MSM, among whom the incidence has been estimated to be 30–100 times the general population [9], [11], [12], [13]. Although early-stage SCCA is readily treatable with chemotherapy and radiation, in the U.S., 5-year survival is about 80% for those with localized disease and about 20% for individuals with metastatic disease [5].

Despite its increasing incidence and the morbidity and mortality associated with this disease, there are still no national guidelines on screening for SCCA among high-risk populations [3], [4], [14], [15]. Cytologic screening and/or HPV DNA testing for SCCA could potentially reduce both incidence and mortality by allowing for early detection and treatment of its associated precursor lesions, high-grade anal intraepithelial neoplasia (HGAIN). The etiologic and biologic similarities between cervical and anal cancer [16] suggest that cervical cancer screening guidelines could be used as a model for anal cancer screening for high-risk populations [17], [18]. Although no randomized clinical trials have been conducted to verify the efficacy of anal cancer screening strategies, some evidence suggests that serial anal cytology testing is a sensitive method for detection of HGAIN [4], [19]. Furthermore, there is evidence that routine anal cytology, high resolution anoscopy (examination under magnification of the anal canal, similar to colposcopy), and treatment of HGAIN would be a cost-effective method of SCCA prevention among selective high risk populations, such as HIV-infected MSM [17], [18].

Until very recently, few geographic areas have had multiple providers routinely offering anal dysplasia screening programs. However, providers in San Francisco, California have been at the forefront of providing anal dysplasia screening targeted towards high-risk populations. For example, there have been five providers in the San Francisco area practicing for the last decade [20]. In particular, the Anal Dysplasia Clinic at the University of California, San Francisco (UCSF) Cancer Center was the first clinic to conduct clinical research on anal cytology screening. This clinic has not only been one of the few clinics in the U.S. that has offered routine anal cancer screening since approximately 1991, but has also provided training in anal dysplasia screening and has helped set the precedent for anal dysplasia screening and treatment in the U.S. (J.M. Berry; personal communication; July 6, 2011). In order to determine whether such a screening program allows for earlier detection of abnormalities and in turn, improves patient survival, we conducted an ecological study using data from the Surveillance, Epidemiology, and End Results (SEER) program of the National Cancer Institute. Specifically, we compared SCCA incidence and survival over time (1996–2008) in the San Francisco-Oakland SEER catchment area (SF-O) to other SEER regions from the rest of California and the rest of the United States in which routine screening has not been as readily available. Additionally, we provided analyses from the pre-HAART era (1985–1995) for comparison.

## Methods

### Study Population: SEER Data

The SEER program provides publicly available cancer incidence and survival data for approximately 28% of the U.S. population [21]. We utilized the most recent SEER dataset (1973–2008, released April 2011), which included the following registries (with the years at which data collection began): Connecticut (1973), Detroit (1973), Hawaii (1973), Iowa (1973), New Mexico (1973), San Francisco-Oakland (1973), Utah (1973), Arizona (1973), Seattle-Puget Sound (1974), Atlanta (1975), rural Georgia (1978), Los Angeles (1992), San Jose-Monterey (1992), Alaska (1999), Greater California (2000), Kentucky (2000), Louisiana (2000), and New Jersey (2000). For the years 1996–2008, SF-O was compared to the other three California registries (Los Angeles, San Jose-Monterey, and Greater California) and to all remaining SEER sites. However, as SF-O was the only California SEER site that started prior to 1992, we could not compare it to the other California sites for the years 1985–1995, and instead compared to all other registries for this time period.

From the full SEER dataset, we restricted our case definition to individuals with first primary squamous cell cancer (ICD-O-3 8010-8089) at anatomic sites coded C21.0, C21.1, and C21.8 (anus, anal canal, and anorectum). Data on date of diagnosis, stage at diagnosis (*in situ*, localized/regional direct extension, distal metastasis), radiation therapy, surgery, patient demographics, follow-up time, and cause-specific mortality was obtained. Cancer-directed treatment was defined as any cancer-directed surgery or radiation therapy.

Because of the major changes over time due to the rise of the HIV/AIDS epidemic in the early 1980s and the development of HAART [19], cases diagnosed between 1985 and the initiation of the HAART era (1996) were analyzed separately for comparison to the post-HAART, post-screening years between 1996 and 2008.

### Statistical Analysis

All analyses were conducted separately for the pre- and post-HAART eras (1985–1995 and 1996–2008). Demographics of SCCA cases, as well as tumor characteristics, were compared between cases by reporting registry site, using Χ^2^ tests. Both crude and age-standardized annual incidence rates were calculated and graphed over time. Population standards were obtained from U.S. Census data, and direct standardization was used to age-standardize to the 2000 U.S. population. Logistic regression was used to determine which factors were associated with the probability of having an *in situ* versus invasive tumor among the SCCA cases. Variables included in the model were registry site, age, race, year of diagnosis, and sex.

Kaplan-Meier survival curves were constructed to visualize differences in survival probability over time by characteristics of interest, and log-rank tests were used to determine whether these differences were statistically significant (α = 0.05). Cox regression models were utilized to evaluate the impact of being in the SF-O region, compared to the rest of California and/or other SEER sites (where routine screening has not been as widely available), on cause-specific mortality hazard over time. Covariates included in the multivariable Cox model were those that were unlikely to be directly involved in the causal pathway that explains how screening could impact survival. These variables were age at diagnosis, diagnosis year, sex, race, and whether the patient received cancer-directed treatment after diagnosis. An additional model adjusting for stage at diagnosis was also run for comparative purposes. However, it should be noted that including stage in the model may obfuscate the pathway determining how routine screening influences survival. All statistical analyses were conducted in SAS version 9.0 (Cary, NC) and STATA 12 (College Station, TX).

## Results

Approximately 15.2% (n = 1602) of the SCCA cases reported to SEER between 1996 and 2008 were from the SF-O region, whereas a higher proportion (24.3%, n = 428) were reported from SF-O between 1985 and 1995. The distributions of the demographic characteristics examined were significantly different between SF-O and the other SEER registries (Tables 1 & 2). Cases reported from the SF-O registry were more likely to be younger and male, whereas cases from other registries had a more equal sex distribution and tended to be older. The majority of cases across all registries were non-Hispanic white. For both pre- and post-HAART eras, lesions diagnosed in the SF-O region were more likely to be *in situ* and were more commonly tumors of the anus, rather than of the anorectum or anal canal.

**Table 1:** Population characteristics of anal cancer cases from SEER dataset: 1996–2008.

Characteristic		SF-O Registry (n = 1602)n (%)	Other CA Registries^a^ (n = 3992)n (%)	All Other Registries (n = 4952)n (%)	p-value
Age					<.0001
	*≤35 years*	175 (10.9)	278 (7.0)	300 (6.1)	
	*36*–*45 years*	524 (32.7)	825 (20.7)	942 (19.0)	
	*46*–*55 years*	467 (29.2)	1080 (27.1)	1401 (28.3)	
	*56*–*65 years*	248 (15.5)	826 (20.7)	963 (19.5)	
	*>65 years*	188 (11.7)	983 (24.6)	1346 (27.2)	
Sex					<.0001
	*Male*	1243 (77.6)	2083 (52.2)	2127 (43.0)	
	*Female*	359 (22.4)	1909 (47.8)	2825 (57.1)	
Race					<.0001
	*Non-Hispanic White*	1108 (69.2)	2863 (71.7)	3870 (78.2)	
	*Black*	181 (11.3)	268 (6.7)	702 (14.2)	
	*Hispanic*	155 (9.7)	566 (14.2)	240 (4.9)	
	*Asian and other*	158 (9.8)	295 (7.4)	140 (2.8)	
Tumor Site					<.0001
	*Anus*	886 (55.3)	1664 (41.7)	2338 (47.2)	
	*Anal canal*	625 (39.0)	1847 (46.2)	2056 (41.5)	
	*Anorectum*	91 (5.7)	481 (12.1)	558 (11.3)	
Cancer-Directed Treatmentb,c					<.0001
	*Yes*	1203 (75.9)	3320 (84.5)	4514 (92.3)	
	*No*	383 (24.2)	611 (15.5)	376 (7.7)	
Stage^c^					
*In situ*	*Male*	895 (89.0)	1066 (76.6)	653 (60.0)	<.0001^d^
	*Female*	111 (11.0)	326 (23.4)	435 (40.0)	
	*Total*	1006	1392	1088	
*Localized/Regional Direct Extension (±Regional Lymph Node)*	*Male*	304 (59.7)	816 (40.4)	1234 (38.8)	<.0001^d^
	*Female*	205 (40.3)	1206 (59.6)	1945 (61.2)	
	*Total*	509	2022	3179	
*Distal Lymph Node/Metastasis*	*Male*	32 (48.5)	78 (27.2)	118 (32.0)	<.0001^d^
	*Female*	34 (51.5)	209 (72.8)	251 (68.0)	
	*Total*	66	287	369	

Note. SF-O: San Francisco-Oakland.

a Includes Los Angeles, San Jose-Monterey, and Greater California registries.

b Includes cancer-directed surgery and/or radiation therapy.

c Frequencies do not sum to column heads because of unknown/missing information.

d P-value compares distributions of sex by registry location within each stage.

**Table 2:** Population characteristics of anal cancer cases from SEER dataset: 1985–1995.

Characteristic		SF-O Registry (n = 428) n (%)	All Other Registries (n = 1333) n (%)	p-value
Age				<.0001
	*≤35 years*	65 (15.2)	112 (8.4)	
	*36*–*45 years*	84 (19.6)	194 (14.6)	
	*46*–*55 years*	80 (18.7)	209 (15.7)	
	*56*–*65 years*	89 (20.8)	302 (22.7)	
	*>65 years*	110 (25.7)	516 (38.7)	
Sex				<.0001
	*Male*	268 (62.6)	547 (41.0)	
	*Female*	160 (37.4)	786 (59.0)	
Race				0.02
	*Non-Hispanic White*	315 (73.6)	1072 (80.4)	
	*Black*	59 (13.8)	148 (11.1)	
	*Hispanic*	34 (7.9)	68 (5.1)	
	*Asian and other*	20 (4.7)	45 (3.4)	
Tumor Site				<.0001
	*Anus*	287 (67.1)	709 (53.2)	
	*Anal canal*	92 (21.5)	386 (29.0)	
	*Anorectum*	49 (11.5)	238 (17.9)	
Cancer-Directed Treatmentb,c				0.26
	*Yes*	403 (94.2)	1234 (92.6)	
	*No*	25 (5.8)	99 (7.4)	
Stage^c^				
*In situ*	*Male*	103 (85.1)	134 (60.1)	<.0001^d^
	*Female*	18 (14.9)	89 (39.9)	
	*Total*	121	223	
*Localized/Regional Direct Extension (±Regional Lymph Node)*	*Male*	136 (54.8)	312 (37.5)	<.0001^d^
	*Female*	112 (45.2)	521 (62.6)	
	*Total*	248	833	
*Distal Lymph Node/Metastasis*	*Male*	7 (58.3)	26 (35.1)	0.20d,e
	*Female*	5 (41.7)	48 (64.9)	
	*Total*	12	74	

Note. SF-O: San Francisco-Oakland.

a Includes Los Angeles, San Jose-Monterey, and Greater California registries.

b Includes cancer-directed surgery and/or radiation therapy.

c Frequencies do not sum to column heads because of unknown/missing information.

d P-value compares distributions of sex by registry location within each stage.

e Fisher's exact test.

**Figure 1:**
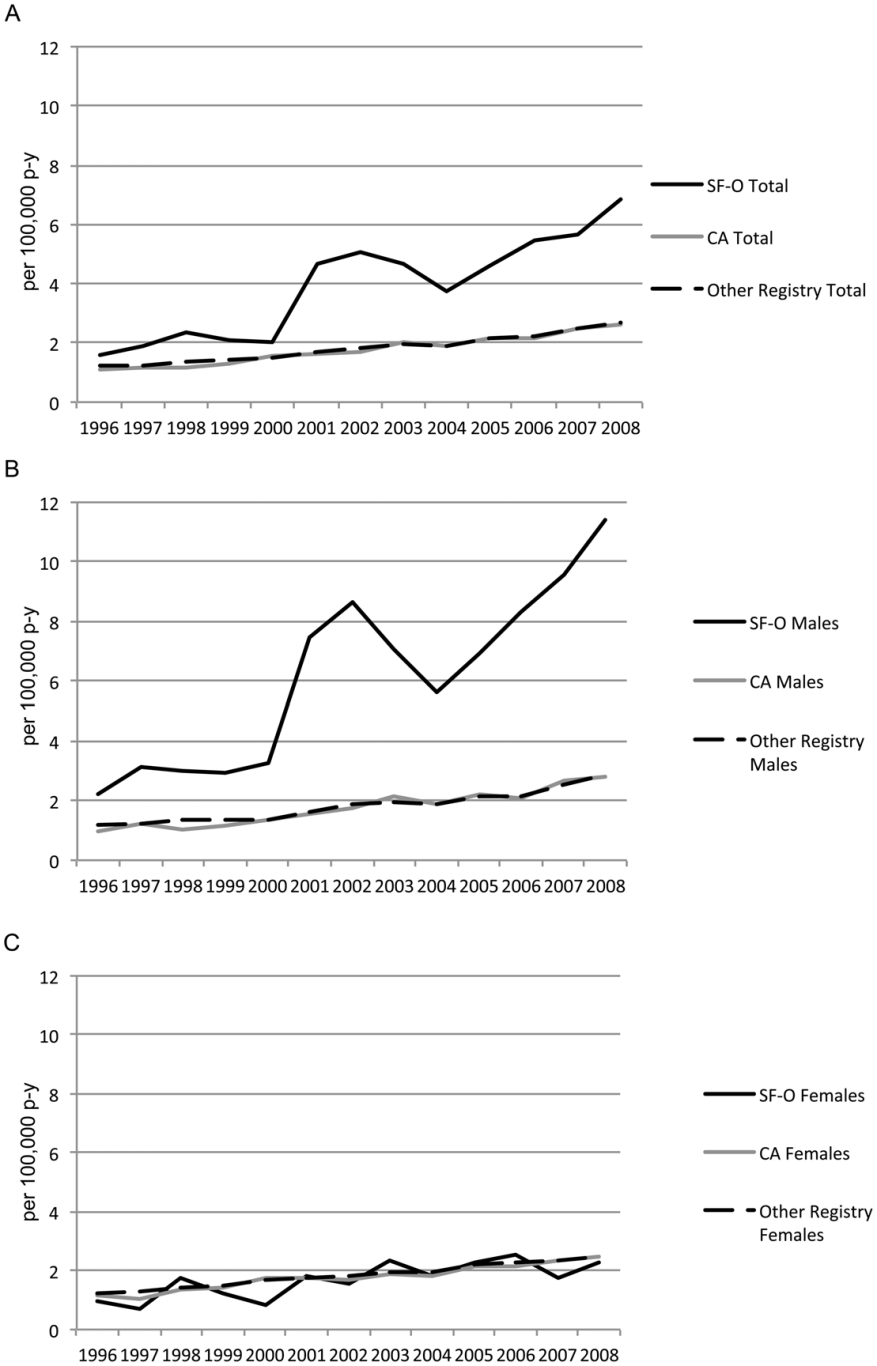
Age-standardized incidence* of squamous cell carcinoma of the anus, anal canal, and anorectum between 1996 and 2008 by sex and SEER registry site, San Francisco-Oakland (SF-O), other California sites (CA), and all other registries. A. Overall; B. Among Men; C. Among Women. Footnote: *Includes *in situ* cases. X-axis: Year. Y-axis: per 100,000 person-years.

**Figure 2:**
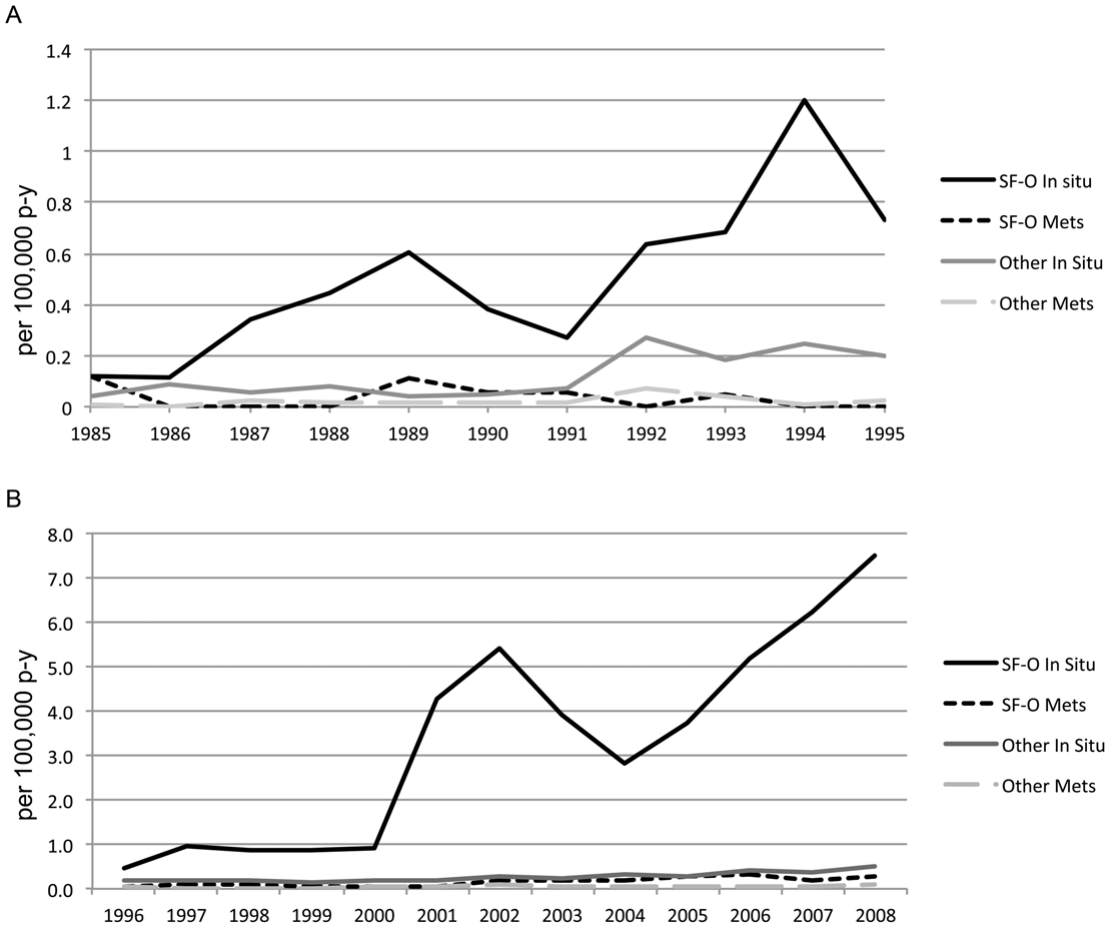
Incidence of *in situ* and metastatic squamous cell carcinoma of the anus, anal canal, and anorectum among men in San Francisco-Oakland (SF-O) compared to all other SEER sites. A. Pre-highly active antiretroviral therapy era (HAART); B. Post-HAART. X-axis. Year. Y-axis: per 100,000 person-years.

Figure 1A–C shows the age-standardized annual incidences of SCCA between 1996 and 2008 by registry site and sex. Overall, there has been a consistent increase in incidence of SCCA over time. Although the increased incidence among women is subtle, there was a substantially increased incidence among men in SF-O. This pronounced increase among men was partially driven by an increase in the number of male *in situ* cases reported from the SF-O region (Figure 2).

Among SCCA cases diagnosed in the post-HAART era, being reported by the SF-O registry was associated with a four fold higher probability of having an *in situ* tumor (as opposed to an invasive tumor) [95% CI: 3.48–4.60; Table 3]. Younger age was also strongly associated with the probability of having *in situ*, rather than invasive, SCCA (OR for ≤35 years old: 17.51, 95% CI: 13.87–22.11, compared to >65 years of age). In the post-HAART era, race/ethnicity was significantly associated with the odds of having *in situ* tumors. Being non-Hispanic white conferred the highest probability of having *in situ* rather than invasive SCCA, compared to other racial/ethnicity categories. Among cases diagnosed between 1985 and 1995, being reported by the SF-O registry was associated with a 41% higher risk of having an *in situ*, rather than invasive, tumor (95% CI: 1.06–1.89; Table 4).

**Table 3:** Multivariable Logistic Regression Model Among All Cases Examining the Impact of Geographic Location on Probability of Having an *In situ* Cancer (Versus Invasive): 1996–2008.

	Adjusted Odds Ratio (95% Confidence Interval)
Geographic Location (Registry site)	
*San Francisco-Oakland*	4.00 (3.48–4.60)
*Other CA Registries*	1.77 (1.59–1.98)
*All Other Registries*	ref
Sex	
*Male*	2.54 (2.29–2.82)
*Female*	ref
Race	
*Non-Hispanic Black*	0.38 (0.31–0.47)
*Hispanic*	0.33 (0.26–0.42)
*Asian and Other*	0.43 (0.34–0.56)
*Non-Hispanic White*	Ref
Year of Diagnosis	1.10 (1.08–1.11)
Age	
*≤35 years*	17.51 (13.87–22.11)
*36*–*45 years*	5.42 (4.62–6.35)
*46*–*55 years*	2.41 (2.07–2.81)
*56*–*65 years*	1.33 (1.12–1.58)
*>65 years*	ref

**Table 4:** Multivariable Logistic Regression Model Among All Cases Examining the Impact of Geographic Location on Probability of Having an *In situ* Cancer (Versus Invasive): 1985–1995.

	Adjusted Odds Ratio (95% Confidence Interval)
Geographic Location (Registry site)	
*San Francisco-Oakland*	1.41 (1.06–1.89)
*All Other Registries*	ref
Sex	
*Male*	1.72 (1.29–2.31)
*Female*	ref
Race	
*Non-Hispanic Black*	0.97 (0.66–1.43)
*Hispanic*	1.54 (0.93–2.56)
*Asian and Other*	1.62 (0.84–3.11)
*Non-Hispanic White*	ref
Year of Diagnosis	1.04 (0.99–1.08)
Age	
*≤35 years*	8.46 (5.36–13.36)
*36–45 years*	5.33 (3.54–8.02)
*46–55 years*	1.96 (1.27–3.03)
*56–65 years*	1.38 (0.89–2.14)
*>65 years*	ref

**Figure 3:**
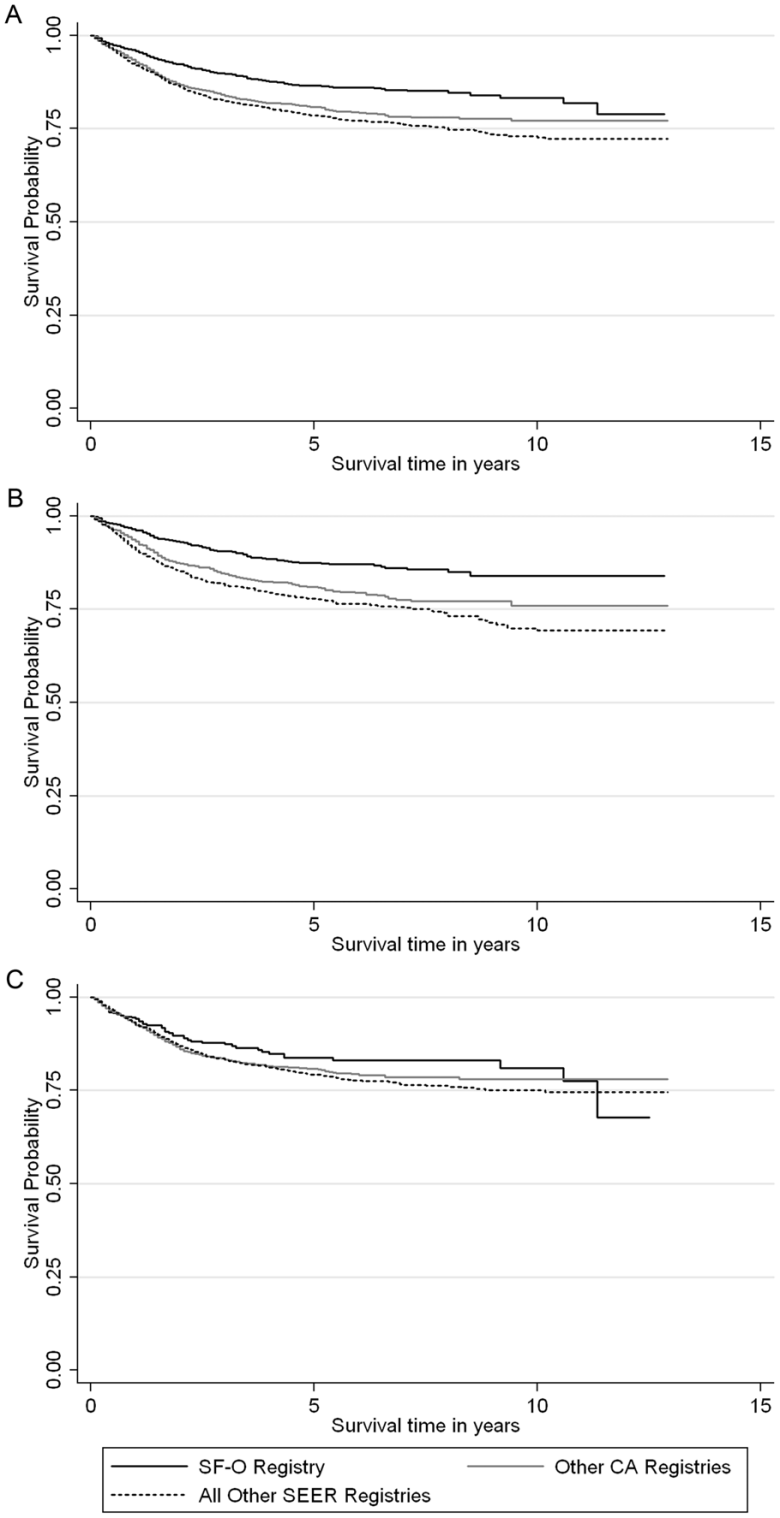
Kaplan-Meier curves comparing cause-specific* survival distributions between the San Francisco-Oakland Registry (SF-O), other California registries (CA), and all other SEER registries (1996–2008), both overall and by sex: A. Overall by registry site (log rank p<0.0001); B. Among males by registry site (log rank p<0.0001); C. Among females by registry site (log rank p = 0.21). Footnote: *All reported cases, including *in situ*, were included in these survival curves.

**Figure 4:**
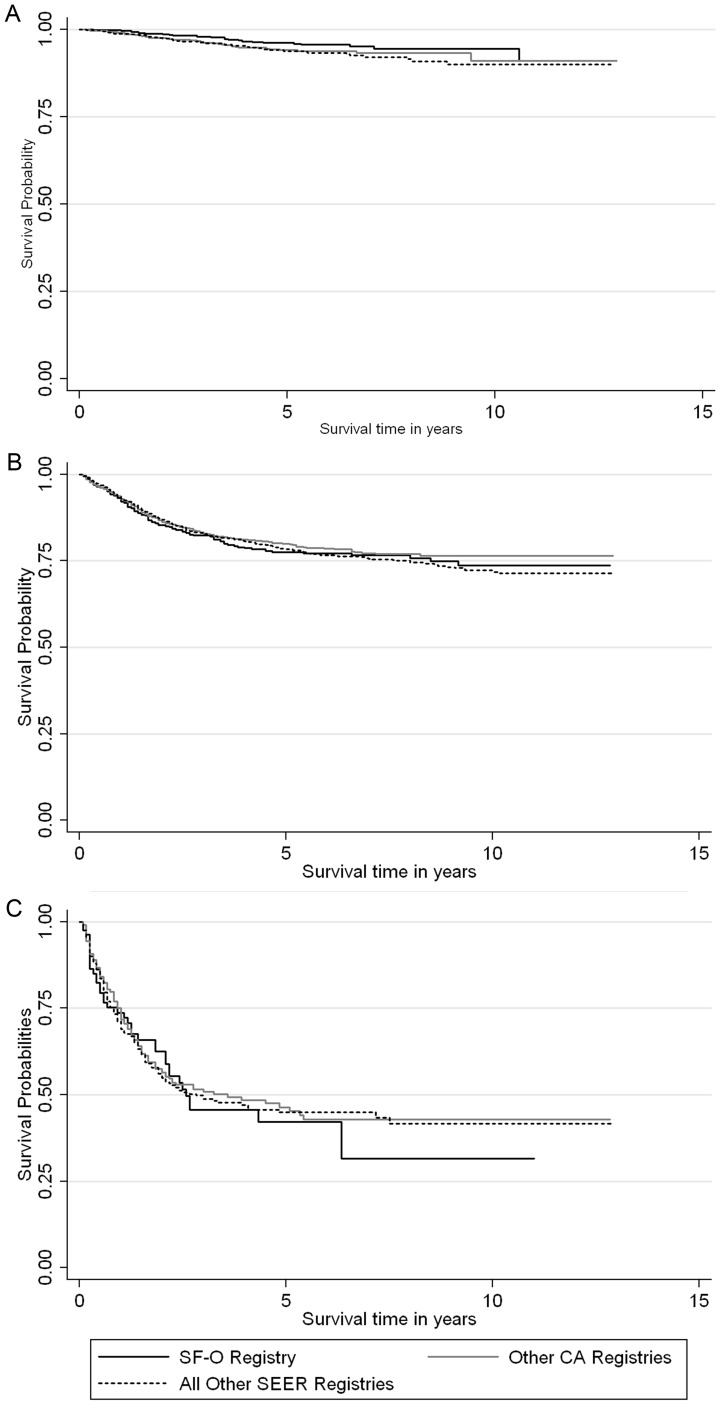
Kaplan-Meier curves comparing cause-specific survival distributions among cases with *in situ* (A), locally/regionally extended (B), and metastic (C) tumors between the San Francisco-Oakland Registry (SF-O), other California registries (CA), and all other SEER registries (1996–2008).

Cases reported by the SF-O registry between 1996 and 2008 had better survival over time than those reported from elsewhere (Figure 3). This differential survival probability was particularly prominent among males (Figure 3B; log rank p<0.0001). However, the survival advantages of SF-O-reported cases were not observed among cases diagnosed between 1985 and 1995 [data not shown]. Additionally, Figure 4 illustrates that regardless of SEER site, stage at diagnosis is a significant predictor of survival over time.

The results of the multivariable Cox regression models examining the association between registry site and mortality hazard, with and without adjustment for stage at diagnosis, are given in Tables 5 & 6. In the post-HAART era, cases reported from the SF-O region had a 39% lower mortality risk than those located in a registry area outside of California (95% CI: 0.51–0.72), controlling for age at diagnosis, year of diagnosis, sex, race, and cancer-directed treatment. Further adjustment for stage resulted in an attenuation of this protective effect (HR for SF-O: 0.85, 95% CI: 0.72–1.02). Additionally, non-Hispanic black race was associated with a higher mortality hazard, regardless of adjustment for stage at diagnosis. In the pre-HAART era, cases reported from the SF-O region had a slightly higher mortality risk compared to cases reported from other sites, regardless of adjustment for stage, but this association was not statistically significant. Male sex and older age at diagnosis were associated with an increased mortality risk in both pre- and post-HAART models.

**Table 5:** Multivariable Cox Regression Models Examining the Impact of Geographic Location on Anal Cancer Mortality: 1996–2008.

	Model Excluding Stage	Fully Adjusted Model
	Hazard Ratio (95% Confidence Interval)	Hazard Ratio (95% Confidence Interval)
Geographic Location (Registry site)		
*San Francisco-Oakland*	0.61 (0.51–0.72)	0.85 (0.72–1.02)
*Other CA Registries*	0.93 (0.82–1.03)	0.98 (0.87–1.10)
*All Other Registries*	ref	ref
Age at diagnosis (years)	1.03 (1.03–1.04)	1.02 (1.02–1.02)
Year of diagnosis	0.97 (0.96–0.99)	0.99 (0.97–1.01)
Sex		
*Male*	1.32 (1.19–1.48)	1.70 (1.52–1.90)
*Female*	ref	ref
Race		
*Non-Hispanic Black*	1.71 (1.48–1.97)	1.65 (1.41–1.92)
*Hispanic*	1.03 (0.86–1.24)	1.07 (0.88–1.31)
*Asian and Other*	0.64 (0.47–0.87)	0.87 (0.64–1.18)
*Non-Hispanic White*	ref	ref
Cancer-Directed Treatment		
*Yes*	0.60 (0.52–0.70)	0.42 (0.35–0.50)
*No*	ref	ref
Stage		
*Distal Lymph Node Involved/Metastasis*	-	21.02 (16.83–26.26)
*Local/Regional Extension*	-	5.35 (4.36–6.56)
*In situ*	-	ref

**Table 6:** Multivariable Cox Regression Models Examining the Impact of Geographic Location on Anal Cancer Mortality: 1985–1995.

	Model Excluding Stage	Fully Adjusted Model
	Hazard Ratio (95% Confidence Interval)	Hazard Ratio (95% Confidence Interval)
Geographic Location (Registry site)		
*San Francisco-Oakland*	1.11 (0.92–1.34)	1.15 (0.94–1.42)
*All Other Registries*	ref	ref
Age at diagnosis (years)	1.01 (1.01–1.02)	1.01 (1.00–1.02)
Year of diagnosis	0.98 (0.96–1.01)	0.98 (0.95–1.01)
Sex		
*Male*	1.58 (1.32–1.90)	1.83 (1.50–2.23)
*Female*	ref	ref
Race		
*Non-Hispanic Black*	1.46 (1.15–1.85)	1.25 (0.96–1.62)
*Hispanic*	0.88 (0.61–1.29)	0.97 (0.65–1.43)
*Asian and Other*	0.83 (0.52–1.32)	0.93 (0.57–1.52)
*Non-Hispanic White*	ref	ref
Cancer-Directed Treatment		
*Yes*	0.41 (0.32–0.54)	0.36 (0.26–0.52)
*No*	ref	ref
Stage		
*Distal Lymph Node Involved/Metastasis*	-	8.11 (5.78–11.38)
*Local/Regional Extension*	-	1.45 (1.13–1.87)
*In situ*	-	ref

## Discussion

To our knowledge this is the first ecological study to explore whether access to anal cancer screening programs may help improve patient survival by allowing for earlier detection and treatment of pre-malignant anal lesions. We specifically compared the incidence and outcomes of SCCA from other SEER registries in the U.S. to the SF-O SEER registry, because unlike other SEER-associated geographic areas, SF-O has several anal dysplasia clinics [20], including the anal dysplasia clinic at UCSF, one of the most established anal dysplasia clinics in the U.S. Our results indicated that a large proportion of cases reported from the SF-O region had *in situ* SCCA at the time of diagnosis. In addition, the incidence rates of SCCA increased in all three areas; thus, we did not find that SCCA screening decreased incidence in SF-O. However, we found that being reported by the SF-O registry was significantly protective against SCCA mortality, compared to being reported from other SEER registries both inside and outside of California. Our study is one of the first to attempt to investigate what potential impact anal cancer screening programs have on patient survival.

Our results indicated that the incidence of SCCA has continually increased since 1996 in both SF-O and the U.S. in general. Although the incidence of invasive SCCA has not decreased, the higher incidence of SCCA observed in the SF-O region (Figure 1) is largely explained by increased rates of *in situ* SCCA among men (Figure 2). In fact, the incidence of *in situ* lesions among men in SF-O is much higher than in other SEER registries, but this higher observed incidence is likely due to detection bias. Because of the UCSF program, men in this area may be more likely to be screened (or targeted for screening), which in turn would increase the probability that *in situ* lesions would be detected and reported to SEER from the SF-O registry. However, this finding is notable also because HIV infection has been associated with an increased risk of SCCA, and the incidence of HIV/AIDS in San Francisco county is one of the highest incidences of HIV compared to most other SEER sites [22].

Furthermore, we also found that overall survival for all SCCA was significantly longer for those reported from the SF-O registry in the HAART era (1996–2008). Particularly, the Kaplan-Meier survival curves illustrate that SF-O patients, especially male patients, have better survival, but this likely correlates with the fact that diagnosis of *in situ* SCCA is associated with younger age (≤35 years) and male gender in the multivariable model. Unlike the overall survival curves that include all SCCAs, the stage-specific survival of SF-O patients in the HAART era is similar to that of patients from other geographic regions, implying that the effectiveness of received treatment for invasive SCCA is similar across the country (Figure 4). Thus, the observed survival difference seen through the Cox regression model (unadjusted for stage) may be due to earlier-stage detection and diagnosis of SCCA in the SF-O region. The improved survival could also be due to lead-time bias which is often associated with cancer screening. Depending on the magnitude of this bias, it is still possible that routine anal cancer screening may confer an important opportunity to detect and treat early stage disease, and consequently, reduce SCCA mortality. This is exactly the paradigm upon which cervical cancer screening programs are based.

Previous research has shown that enabling factors, such as the presence of screening programs, are associated with the intention to seek anal cancer screening among high-risk populations. Specifically, D'Souza et al. found that MSM who believed that anal cancer screening was available in their community had a 2.2 times higher likelihood of intending to get screened within the next 6 months, even after adjustment for various demographic, biological, and behaviorial factors (95% CI: 1.7–2.8) [15]. Given that intention to seek a preventive health service has been shown to be strongly associated with subsequent use of such service, the availability of regional screening programs may be a strong predictor of participation in anal cancer screening among high risk groups.

One key limitation of our study was that individual-level data on participation in anal cancer screening was not available. Thus, we cannot state with certainty that the improved survival observed in the SF-O region is attributable to the availability of routine screening in the area. There is a possibility that all SEER sites may not have standardly reported the pathologic diagnosis of “in situ” cases, as opposed to anal intraepithelial neoplasia 3. As with any study in which examination of tumor samples by a study pathologist is not posssible, there is potential misclassification with regard to pathology. Because of this, the results presented here, particularly with regard to *in situ* tumors, should be interpreted with caution.

Additionally, when we controlled for stage at diagnosis in the Cox models, the protective effect associated with SF-O in the post-HAART era became attenuated and was no longer statistically significant. Earlier stage at diagosis is in the causal pathway between screening (and consequent early detection) and survival, but this does not discount the strong possibility that the survival benefit associated with the SF-O area may simply be due to lead time bias, particularly in the context of the distinct demographic profile of the SF-O cases. Thus, another limitation of this study is that the observed survival differential may be attributable to other differences between the SF-O patient population and the populations of other SEER sites. Cases from SF-O were generally younger than cases reported from other SEER sites. Although we cannot entirely account for residual confounding by age on the association between SEER site and survival, *post-hoc* survival analyses stratified by age indicated that being reported from the SF-O registry was generally associated with a protective effect in both younger and older subsets of cases. We also did not have information on HIV status of cancer cases and thus could not account for that in our models. However, we divided our analysis by the pre-HAART and the post-HAART eras, to partially determine the effect of HIV infection on SCCA incidence in the SEER registries. In addition, we adjusted for factors for which we had available data, but we did not have individual-level information on health care utility, socioeconomic status, baseline health status, and other such characteristics. Ideally, analyses that account for competing risks should be conducted in future studies that are equipped to do so in order to better clarify the potential survival benefit due to screening and early detection. We cannot conduct such analyses with the SEER dataset, as no information is available on comorbidities or other relevant variables. Nevertheless, this is the first and only study, to our knowledge, to capitalize on this large, publicly available dataset to attempt to study the role of screening in anal cancer prognosis.

Despite the increasing incidence of SCCA in the U.S. and other developed countries [5], [6], [7], there are few providers who offer routine anal cancer screening. The lack of providers may partially be explained by the fact that there is little available evidence on the potentially beneficial impact of routine screening among high risk populations and no national screening guidelines for SCCA [3], [14]. However, given the high prevalence of high-grade AIN among MSM and HIV-positive individuals [23], it is surprising that a randomized-controlled trial has not yet been conducted to show how screening may impact patient prognosis. In the absence of such a clinical trial, our results, in combination with the previous cost-effectiveness studies [17], [18], provide preliminary evidence that routine anal cancer screening programs could potentially help detect SCCA at an earlier stage. Therefore, the impact such programs may make on patient survival warrants additional study.
